# The effective and innovative cost saving of software in managing unregistered drugs

**DOI:** 10.1186/2052-3211-8-S1-P25

**Published:** 2015-10-05

**Authors:** Nur Wahida Zulkifli, Noorizan Abd Aziz, Yahaya Hassan, Mohamed Azmi Hassali, Nur Liyana Zainal Bahrin

**Affiliations:** 1 Department of Pharmacy Practice, Faculty of Pharmacy, University Technology MARA, Bandar Puncak Alam, 42300, Malaysia; 2 School of Pharmaceutical Sciences, Faculty of Pharmacy, University Sains Malaysia, Penang, 11800, Malaysia

## Background

The Annual Report of Enforcement Pharmacy raiding of unregistered drugs has shown an increasing trend since 2010 until 2012 and there is a possibility that the seized value will keep on increasing year by year. Furthermore, the Ministry of Health (MOH) has come out with the Malaysian National Medicines Policy (MNMP): one of the strategies in the policy is the management of counterfeit drugs and one of the solutions is to monitor the retailers and wholesalers through inspection. The main objective of this study is to develop software for managing unregistered drugs through an awareness programme and inspection activities.

## Methods

This is a cross-sectional retrospective study. The data were obtained from the Enforcement Pharmacy Unit, Selangor and the inspection form was created by the Pharmacy Services Division, Malaysia. The premises were divided according to districts and there are 12 districts in Selangor. All of the files were in Microsoft Excel and were divided by month from January until December 2013. Then, data were extracted from Microsoft Excel which included the district of the premises, type of the premises, type of offences, and the survey of the awareness of registered drugs.

## Results

In total, only 474 (32.9%) general retailers are aware about registered drugs and 967 (67.1%) general retailers are not aware about registered drugs. About 12 districts were involved in the survey, and only one district (Hulu Selangor Districts Council) achieved more than 50% awareness about registered drugs. Finally, based on chi-square (0.226), the relationship between awareness and offences is not significant.

## Conclusions

The general retailers areaware of the unregistered health products or even if they are not aware; those retailers would still commit the offences. This might due to the high profit that usually is the aim of the every business in the industry. This result will be used as a pioneering study and as a reference for future study. In addition, the data retrieved from the inspection form will also be used for the development of awareness and inspection software including all the details of geographical location of the premises. Monitoring of the premises by an enforcement officer costs a lot of money because there is no specific database that can be used as a reference and it is very tedious gathering all of the information about the general retailers' premises.

